# A Position Modification Device for the Prevention of Supine Sleep During Pregnancy: A Randomised Crossover Trial

**DOI:** 10.1111/1471-0528.17952

**Published:** 2024-09-16

**Authors:** Danielle L. Wilson, Carley Whenn, Maree Barnes, Susan P. Walker, Mark E. Howard

**Affiliations:** ^1^ Institute for Breathing and Sleep, Austin Health Melbourne Victoria Australia; ^2^ Department of Obstetrics and Gynaecology The University of Melbourne Parkville Victoria Australia; ^3^ School of Electrical Engineering and Computer Science The University of Queensland St Lucia Queensland Australia; ^4^ Department of Medicine The University of Melbourne Parkville Victoria Australia; ^5^ Mercy Perinatal, Mercy Hospital for Women Heidelberg Victoria Australia

**Keywords:** accuracy of self‐report, birthweight, foetal heart rate, night‐to‐night variability, pillow, pregnant, side sleeping, sleep apnoea, sleep‐disordered breathing, stillbirth

## Abstract

**Objective:**

To assess the effectiveness and acceptability of a pillow‐like position modification device to reduce supine sleep during late pregnancy, and to determine the impacts on the severity of sleep‐disordered breathing (SDB) and foetal well‐being.

**Design:**

Randomised cross‐over study.

**Setting and Population:**

Individuals in the third trimester of pregnancy receiving antenatal care at a tertiary maternity hospital in Australia.

**Methods:**

Participants used their own pillow for a control week and an intervention pillow for a week overnight, in randomised order. Sleep position and total sleep time for each night of both weeks were objectively monitored, with a sleep study and foetal heart rate monitoring performed on the last night of each week.

**Main Outcome Measures:**

Primary outcome = percentage of sleep time in the supine position; secondary outcomes = apnoea–hypopnoea index, foetal heart rate decelerations and birthweight centile.

**Results:**

Forty‐one individuals were randomised with data collected on 35 participants over 469 nights. There was no difference in percentage of total sleep time in the supine position overnight between the control or intervention pillow week (13.0% [6.1, 25.5] vs. 16.0% [5.6, 27.2], *p* = 0.81 with a mean difference of 2.5% [95% CI] = −0.7, 5.6, *p* = 0.12), and no difference in the severity of SDB or foetal heart rate decelerations across weeks. However, increased supine sleep was significantly related to a higher apnoea–hypopnoea index (*r*_s_ = 0.37, *p* = 0.003), lower birthweight (*r*_s_ = −0.45, *p* = 0.007) and lower birthweight centile (*r*_s_ = −0.45, *p* = 0.006). The proportion of supine sleep each night of the week varied widely both within and across participants, despite awareness of side‐sleeping recommendations.

**Conclusions:**

We found no evidence to suggest that the adoption of a pillow designed to discourage supine sleep was effective in late pregnancy, with women spending an average of 1 h per night supine. Alternative devices should be investigated, incorporating lessons learnt from this study to inform trials of supine sleep minimisation in pregnancy.

**Trial Registration:**

Clinical Trial: (Australia New Zealand Clinical Trials Registry): ACTRN12620000371998

## 1 Introduction

Late stillbirth, which is the loss of a pregnancy after 28 weeks of gestation, remains a common and devastating human tragedy, even in high‐income countries such as Australia [1]. Many of these tragic deaths remain unexplained. Recent meta‐analyses have shown that reported ‘going‐to‐sleep’ on the back in pregnancy is a major risk factor for late pregnancy stillbirth [2, 3] Women who self‐report supine sleep in late pregnancy also have an increased risk of low birthweight infants [4, 5], which is one of the strongest risk factors for stillbirth [6]. One proposed rationale is that the enlarged uterus causes aortocaval compression, with reduced cardiac output and placental blood supply [7].

More than 80% of pregnant women spend time sleeping on their back, with the overall proportion of supine sleep time ranging from 9 to 26% [8, 9, 10, 11]. As seen in nonpregnant populations, supine sleep is associated with increased severity of sleep‐disordered breathing (SDB) [10, 12], which in turn has been linked with numerous pregnancy complications [13, 14]. During supine sleep in late pregnancy, foetal activity is reduced with an increase in foetal heart rate decelerations, which may indicate an adaptive behavioural state in response to mild hypoxic stress [9, 15]. Maternal sleep apnoea and/or foetal responses to impaired placental blood flow may, thus, both be important intermediaries in the relationship between supine sleep and stillbirth risk.

Maternal health professionals in Australia, New Zealand and the United Kingdom now promote improved awareness of maternal safe sleeping positions [16, 17, 18], yet ensuring a lateral position is maintained during sleep can be difficult. Also, some controversy on the relationship between supine sleep and stillbirth exists [19, 20], and the effectiveness of public health advice regarding side sleeping on foetal well‐being awaits objective evaluation.

So far only one device for pregnant women has been clinically tested to avoid ‘back‐sleeping’ with single‐night success [9, 21] however, a 12‐week trial showed that it was ineffective at reducing supine sleep with poor compliance and no benefit for the health of the baby [22]. An effective sleep position modification device which is acceptable to heavily pregnant women is needed.

The primary aim of this randomised crossover trial was to assess a pillow‐like position modification device designed to reduce the amount of time pregnant women spend sleeping supine. Secondly, we aimed to assess whether the device would impact on sleep quality and be acceptable to the user. If the device successfully reduced supine sleep during pregnancy, we hypothesised that SDB severity, antenatal indicators of foetal well‐being (foetal heart rate monitoring) and birthweight centile would be improved with its use.

## 2 Method

### 2.1 Study Design and Participants

This study was a single‐centre, randomised crossover repeated measures study, investigating the efficacy of an intervention pillow to reduce supine sleep in late pregnancy, conducted between December 2020 and November 2021. Participants were recruited from the antenatal outpatient clinics, aged 18 years or older with a singleton pregnancy in the third trimester (> 28 weeks of gestation) without known foetal abnormalities or pregnancy complications. The Human Research Ethics Committee at the Mercy Hospital for Women approved the study and written informed consent was obtained from all participants. There was no patient or public involvement in the development of this study, although public health messaging was actively promoting side sleeping during pregnancy.

### 2.2 Intervention and Procedure

For a full description of the study methods, see Appendix S1. The study design involved two study weeks; (1) the intervention week involved the participant using the ‘Back‐Off’ pillow for seven consecutive nights, and (2) the control week involved the participant using their own pillows for seven consecutive nights with no additional advice given regarding sleeping position. The participants were 1:1 block‐randomised (in blocks of 4) with half assigned to complete the intervention week first, and the other half assigned to complete the control week first by DW via Study Randomizer [23]. The first study week was commenced between 30 and 36 weeks of gestation. After a 1‐week ‘wash‐out’ period, each participant crossed over to complete the other arm of the study.

The sleep position modification device was the ‘Back‐Off’ pillow (Figure S1), which is a U‐shaped pillow designed to prevent supine sleep. One arm of the pillow is soft and designed to support the arms and abdomen, whereas the other arm is firm and rests behind the back, to prevent rolling onto the back. The pillow can be flipped over to use lying on either side. A Velcro strap under the pillow holds the arms together to stop them from splaying apart.

On each of the seven nights of the intervention and control week, the participant's body position was monitored with the Night Shift Sleep Positioner (Advanced Brain Monitoring, Carlsbad, CA). This match‐box‐sized device is worn at the back of the neck and held in place with a silicon rubber strap. It is an intervention to deter supine sleep for people with obstructive sleep apnoea and is designed to provide vibro‐tactile feedback when the supine position is detected; this setting remained off for all participants. A three‐axis accelerometer is used to determine neck position (upright, supine, lateral left, lateral right and prone) and to perform an actigraphy‐based classification of sleep versus wake [24].

A sleep diary (paper or electronic) was completed each morning to record perceived sleep duration, sleep quality and sleep position for each night. For the intervention week, questions regarding the use and comfort of the Back‐Off pillow were included. During the control week, questions were asked about the use of additional pillows.

On the last night (night 7) of each week, the participants completed an in‐home self‐applied sleep study with the WatchPAT 300, with concurrent foetal heart rate (cardiotocography; CTG) monitoring using the Monica AN24. The WatchPAT 300 (Itamar Medical, Israel) is a wrist‐worn device with a plethysmographic‐based finger probe and a sensor adhered to the chest right under the sternal notch, which measures peripheral arterial tone (PAT), oxygen saturation (SpO_2_), heart rate, movement, body position, snoring and chest motion. PAT apnoea–hypopnoea index (pAHI) was defined as the number of apnoeas and hypopnoeas per hour of sleep. Snoring was evaluated by an acoustic decibel detector in the chest sensor [25]. Sleep and wake were identified with the built‐in actigraph. Among pregnant women, the WatchPat 300 demonstrates excellent sensitivity and specificity for identification of obstructive sleep apnoea, particularly for pAHI ≥ 5 [26].

CTG was performed using the Monica AN24 (Monica Healthcare Ltd.) portable monitor, as previously described [27] and in Appendix S1. The data were downloaded and scored by the internal algorithm. Each CTG was reviewed by an obstetrician when the monitor was returned to ensure no clinical action was required.

Initially, participants were given the Back‐Off pillow, Night Shift Sleep Positioner and WatchPAT300 in person to self‐apply at home and were set up with the Monica AN24 by a researcher at the hospital. However, during July to November 2021, equipment was provided via noncontact drop‐offs to their homes due to COVID‐19 lockdowns. During this period, researchers were not permitted to meet face‐to‐face so participants were given detailed instructions to attach the Monica AN24 foetal heart rate monitor themselves.

Following completion of both study arms, participants completed a sleep position questionnaire which included questions on whether they had received any advice regarding sleep position in pregnancy.

Basic demographic and obstetric data were collected at recruitment to the study, including maternal age, parity, gestation and body mass index (BMI) at the first antenatal visit. Birth outcomes included birthweight and birthweight centile which was customised for gestational age, maternal height and weight, parity and foetal sex using the GROW software (Bulk Centile Calculator V8.0.6.2; www.gestation.net) [28].

### 2.3 Outcomes

The primary outcome was the percentage of total sleep time in the supine position per night, measured across the intervention and control week with the Night Shift Sleep Positioner. Secondary exploratory outcomes included sleep quality and SDB indices, foetal heart rate decelerations, birthweight and birthweight centile.

### 2.4 Sample Size

Our previous study showed that healthy pregnant women spent a median of 17.7% (IQR 0.7–39.7) of their sleep time in the supine position [29]. Similar to our study, Kember et al. [21] showed that the percentage of supine sleep time on a sham treatment night was 16.4% (IQR 3.5–25.3), compared to 3.5% on the treatment intervention night. Based on this, to identify a reduction of supine sleep time from 16.4% to 3.5% of the night would require a sample size of 26 for a power of 80% at an alpha level of 0.05. Increasing power to 90% would require a sample size of 34, and accounting for potential data loss of 15% of participants similar to that described in Kember et al. and Warland et al. [9] studies, this study aimed for a sample size of 40 participants.

### 2.5 Statistical Analysis

All statistical analyses were performed with Stata 17.0 (StataCorp LP, College Station, TX). Data are given in means with standard deviation (M [SD]) or median and interquartile range (Mdn [IQR]) for nonnormally distributed variables. Mean differences (95% confidence interval) between control and intervention weeks for each of percentage and minutes of supine sleep per night, total sleep time, pAHI and foetal heart rate decelerations were compared to zero (no change) with one‐sample *t*‐tests. A two‐sided *p* value of < 0.05 was considered to indicate statistical significance.

#### 2.5.1 Primary Outcome

To compare the percentage of total sleep time in the supine position per night across study arms, an intention‐to‐treat analysis with linear mixed modelling was conducted to account for variability across nights within each participant (more detail in Appendix S1). Fixed factors were study arm (control vs. intervention week) and night number (1–7), and the random factors were participant and study arm (control vs. intervention week). For the random factor, study arm was nested within each participant. Participants who did not complete both study arms were excluded from the analysis.

#### 2.5.2 Secondary Outcomes

For comparison of sleep quality across weeks, linear mixed modelling was conducted as detailed in Appendix S1. Comparison of SDB indices on the WatchPAT 300 across control and intervention Night 7 was performed using Wilcoxon signed rank sum tests. Due to skewed supine sleep data, Spearman's rank order correlation was used to assess the relationship between supine sleep overnight and SDB, birthweight and birthweight centile. Given the associations between self‐reported supine sleep and birthweight [4, 5], we performed a post hoc exploratory analysis, investigating *objectively confirmed sleep position and birthweight* using control week data only (as a surrogate for normal sleep behaviour in the third trimester), with linear regression used to adjust for gestational age.

The Monica VS software automatically tallies the number of foetal heart rate decelerations per hour of trace, classified as small (a decrease in the foetal heart rate from the baseline ≥ 10 bpm and lasting for ≥ 10 s) and large (a fall in foetal heart rate from the baseline of ≥ 20 bpm and lasting ≥ 60 s). Comparison of foetal heart rate decelerations per hour across control and intervention nights was performed using paired‐sample t‐tests and Wilcoxon signed rank sum tests; hourly blocks with > 25% foetal heart rate trace loss were excluded from the analysis.

To utilise data from all CTGs, linear mixed model analysis was performed to compare hour blocks of CTG recording containing > 50% supine maternal sleep to hour blocks that were predominantly lateral sleep. To do this, maternal sleep position data on the Night Shift Sleep Positioner were synchronised with the CTG recording.

## 3 Results

### 3.1 Participants

**FIGURE 1:**
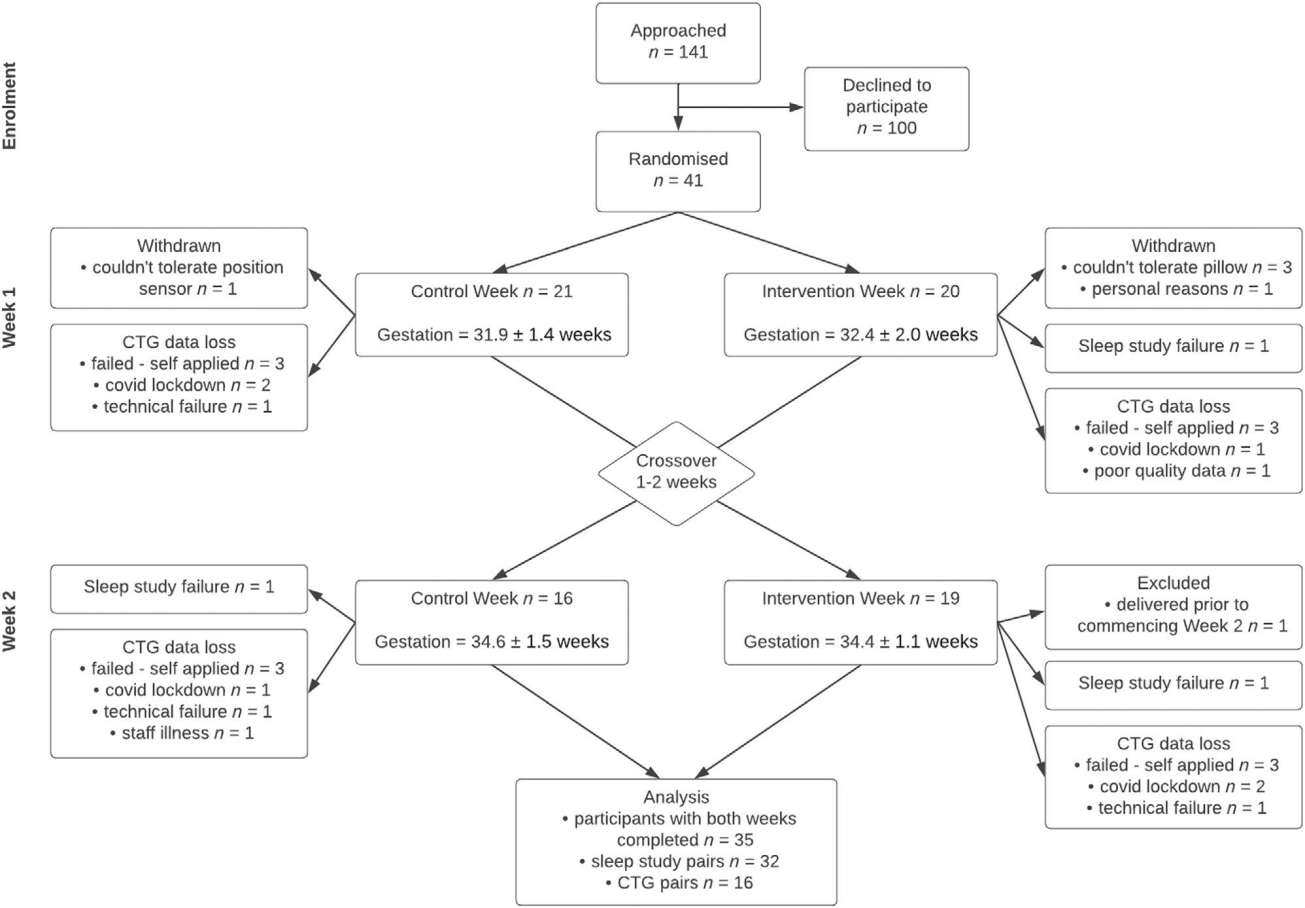
Consort Diagram. During COVID‐19 lockdown, six CTGs were unable to be performed. Following the development of a revised protocol, 27 CTGs were self‐applied at home (15 successes; 12 failures).

Forty‐one women were randomised to the study, and sleep position data for the control and intervention weeks were collected for 35 women over 469 nights (Consort Diagram—Figure 1). The average age of participants was 34.3 (3.2) years, with an average BMI measured at the first antenatal visit of 24.2 (4.1) kg/m^2^. Gestational age at the start of each study week was similar across each arm of the study (Table 1).

**TABLE 1:** Participant characteristics overall and by study arm.

	Overall (*n* = 35)	Control week first (*n* = 19)	Intervention week first (*n* = 16)
Age (years)	34.3 (3.2)	34.4 (3.6)	34.2 (2.8)
BMI at first visit	24.2 (4.1)	23.6 (2.7)	25.0 (5.4)
Gestational age (weeks) — week 1	32.2 (1.7)	32.0 (1.4)	32.4 (2.0)
Gestational age (weeks) — week 2	34.5 (1.3)	34.4 (1.1)	34.6 (1.5)
Nulliparous	19 (54%)	12 (63%)	7 (44%)

*Note:* Values given as *M* (SD) or *n* (%). Characteristics for participants who completed data collection. Six out of 41 women recruited to the study withdrew before completing the first study arm. BMI measured at first antenatal visit (first trimester).

### 3.2 Primary Outcome: Supine Sleep Position Across the Intervention and Control Weeks

**FIGURE 2:**
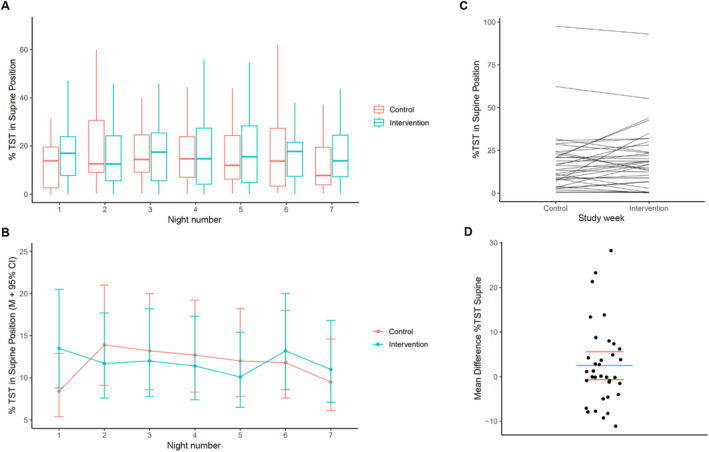
Percentage of total sleep time (TST) spent in the supine position for each night of the week during the control and intervention week as (A) raw data boxplots (extreme outliers removed) and (B) with Mean and 95% confidence intervals with values based on parameter estimates (log‐ then back‐transformed) for the linear mixed model with covariate of gestation. (C) The median nightly percentage of TST per participant for each study week, and (D) The mean difference (blue) with 95% CI (red) for the intervention—control week, with each point representing a participant.

The median percentage of supine sleep per night across the control week was 13.0% (6.1, 25.5) compared to 16.0% (5.6, 27.2) in the intervention week (Figure 2A), with a mean difference of 2.5% (95% CI = −0.7, 5.6, *p* = 0.12; Figure 2C,D). This equates to 53.5 min (23.5, 111.5) per night supine compared to 66.0 min (24.3, 114.0), with a mean difference of 9.8 min (95% CI = −3.8, 23.3, *p* = 0.15). Linear mixed modelling demonstrated that there was no effect of the intervention pillow on percentage of supine sleep overnight (Figure 2B; *p* = 0.81) compared to control, and the percentage of supine sleep per night did not change across the week (*p* = 0.46). Gestational age was included in the model but did not reach significance (*p* = 0.06). All other covariates tested in the model were not significant (*p* > 0.2). The intervention pillow did not reduce the frequency of supine sleep onset (7.3% of nights) compared to the control week (8.7%, χ^2^ (1) = 0.43, *p* = 0.51, risk difference = −1.4%).

During the control week, 42% of women reported using pillows to support behind the back; however, supine sleep overnight was not reduced for these women (12.7% [5.7, 30.2]) compared to those who did not use extra pillows (13.2% [6.3, 23.9], *p* = 0.29). Thirty‐two women completed the sleep position questionnaire; 30 (94%) had received advice regarding safe lateral sleeping position with most reporting they had received advice from their midwife (53.3%) or the internet (56.7%), followed by their obstetrician (20%) and family/friends (20%).

The Night Shift Sleep Positioner was worn for 96% of study nights, for a total of 235 control nights and 234 intervention nights. All‐night adherence (assessed via the sleep diary) with the intervention pillow was high at 88.5%, with partial‐night pillow use for another 8.2% of nights. Only on 3.3% of nights (eight nights), the pillow was not used at all.

**FIGURE 3:**
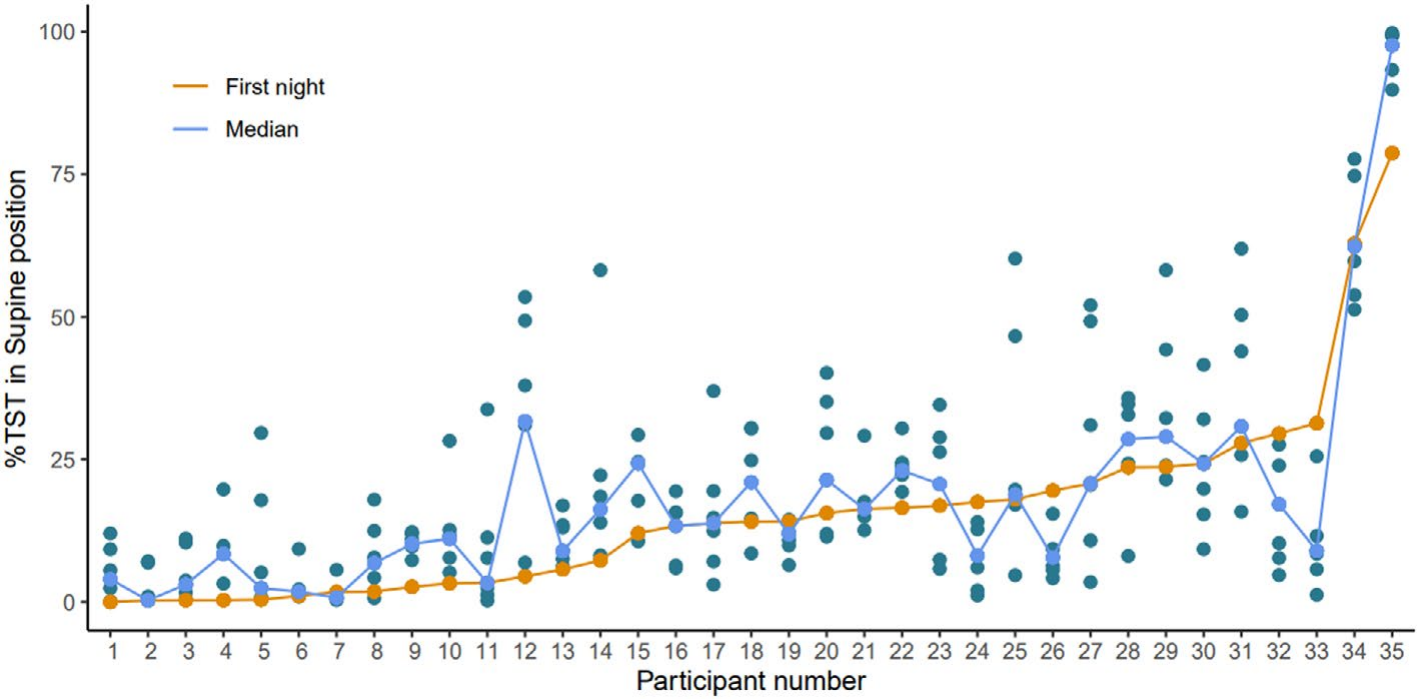
Percentage of total sleep time (TST) spent in the supine position for each participant for each night of the control week (blue dots), with the first night of measurement (orange dots/line) compared to the median (cyan dots/line). The average difference in percentage of supine sleep between the first night of measurement and the median for the week was 5.9% (SD = 6.4), with a relative difference of −2.5% (SD = 8.5) indicating the first night of measurement typically underestimated the overall median. For 11 women (31%), the first night of measurement was their lowest amount of supine sleep for the week. Participants are ordered from lowest to highest first night measurement.

There was wide variability in the percentage of supine sleep per night. In the control week, the average range between lowest and highest supine sleep percentage for the week was 24.4% (13.6). As illustrated in Figure 3, the largest variability was 55.5% between the lowest and highest amount of nightly supine sleep (ID 25), with the most consistent person having a range of only 5.3% across the week (ID 7).

### 3.3 Secondary Outcomes

#### 3.3.1 Perception of Sleep Onset Position

During the control week, women reported their body position at sleep onset correctly on 70.5% of nights, compared to 77.5% of nights during the intervention week. While 37 of 469 nights (7.9%) had objectively recorded supine sleep onset, women reported supine sleep onset in only 3/469 (0.6%) nights.

#### 3.3.2 Sleep Quality

Total sleep time measured by the Night Shift Sleep Positioner was significantly higher in the intervention week compared to the control week (mean [95% CI] difference 14.3 [1.0, 27.6] min, *p* = 0.04; Table 2); however, there was no difference in time spent awake overnight. During the intervention week, there was a preference for left lateral sleep (47.3% vs. 43.1% of the night, *p* = 0.01), compared to more right lateral sleep in the control week (36.6% vs. 31.6% of the night, *p* = 0.009).

**TABLE 2:** Secondary outcomes of sleep quality, indices of sleep‐disordered breathing and foetal heart rate decelerations during the control and intervention week.

	Control week *n* = 35	Intervention week *n* = 35	*p*	Mean difference intervention—Control (95% CI)	*p*
Night Shift Sleep Positioner	*M* (95% CI)	*M* (95% CI)			
Total Sleep Time (min)	409.5 (393.7, 425.4)	421.1 (405.2, 436.9)	0.03	14.3 (1.0, 27.6)	0.04
Sleep Efficiency (%)^a^	83.4 (81.0, 85.6)	84.6 (82.4, 86.6)	0.02	0.9 (−0.3, 2.2)	0.14
WASO mins^a^	65.1 (55.0, 77.1)	61.2 (51.7, 72.5)	0.11	−0.3 (−6.3, 5.7)	0.92
Left %TST	43.1 (37.8, 48.5)	47.3 (41.9, 52.7)	0.01	4.2 (0.04, 8.44)	0.04
Right %TST	36.6 (31.5, 41.7)	31.6 (26.5, 36.7)	0.009	−6.2 (−10.4, −2.0)	0.004
Prone %TST^a^	0.2 (0.1, 03)	0.2 (0.1, 0.2)	0.12	0.04 (−0.08, 0.18)	0.46
Sleep Diary	*M* (95% CI)	*M* (95% CI)			
Sleep Latency (min)^a^	12.8 (9.9, 16.5)	14.2 (11.0, 18.2)	0.33	5.9 (−2.2, 14.0)	0.15
Sleep Duration (min)	436.5 (418.3, 454.8)	432.4 (414.1, 450.7)	0.54	−8.7 (−28.6, 11.2)	0.38
How many times awake?	3.2 (2.6, 3.8)	3.1 (2.5, 3.6)	0.35	0.1 (−0.2, 0.3)	0.59
WASO mins^a^	19.3 (14.4, 25.7)	24.8 (18.6, 33.0)	0.01	2.9 (−3.9, 9.8)	0.39
Quality of sleep out of 10^a^	6.8 (6.4, 7.1)	6.7 (6.3, 7.0)	0.44	−0.1 (−0.4, 0.3)	0.62
SDB indices	*n =* 3*2*	*n =* 32			
Total Sleep Time (min)	402.8 (12.4)	408 (13.4)	0.73	5.8 (−27.9, 39.5)	0.73
pAHI (/h)	1.8 (0.6, 4.4)	1.7 (0.8, 5.4)	0.38	−0.4 (−1.6, 0.9)	0.56
pRDI (/h)	6.9 (2.6, 12.4)	6.0 (4.5, 9.9)	0.91	−0.2 (−2.2, 1.8)	0.85
pODI (/h)	0.4 (0.1, 1.2)	0.5 (0.1, 1.0)	0.52	−0.4 (−1.1, 0.2)	0.19
Mean SpO2	95.8 (0.1)	95.9 (0.2)	0.83	0.03 (−0.3, 0.3)	0.83
Mild snoring (>40 dB) %TST	16.6 (5.6, 64.7)	11.1 (4.8, 78.1)	0.75	0.8 (−12.1, 13.8)	0.90
Supine %TST	9.0 (5.1, 21.2)	13.8 (7.0, 25.9)	0.48	−1.2 (−6.8, 4.4)	0.67
FHR decelerations per hour	*n = 16*	*n = 16*			
Small Decelerations	2.3 (1.2)	3.0 (1.0)	0.03	0.7 (0.1, 1.3)	0.03
Large Decelerations	0.0 (0.0, 0.2)	0.0 (0.0, 0.2)	0.97	−0.01 (−0.18, 0.17)	0.94
Supine %TST	12.5 (6.4, 33.1)	13.4 (3.6, 16.9)	0.06	−6.7 (−14.2, 0.8)	0.08

*Note:* Values for Night Shift Sleep Positioner and sleep diary based on parameter estimates for the linear mixed model. Values for SDB indices and FHR decelerations given as *M* (SD) or Mdn [IQR].

Abbreviations: FHR, foetal heart rate; pAHI, apnoea hypopnoea index on WatchPAT 300; pODI, oxygen desaturation index on WatchPAT 300; pRDI, respiratory disturbance index on WatchPAT 300; SDB, sleep‐disordered breathing; TST, total sleep time; WASO, wake after sleep onset.

^a^ Variable was log transformed for LMM, then back transformed to obtain *M* (95% CI) values.

Based on the sleep diary, women reported no difference in sleep quality between the study weeks apart from more wake after sleep onset in the intervention week (*p* = 0.01; Table 2). The average rating out of 10 for comfort of the pillow was 6.5 (1.5), with two‐thirds reporting that they would continue using the pillow during pregnancy.

#### 3.3.3 Sleep‐Disordered Breathing

There were 32 pairs of WatchPat 300 data available for the control and intervention Night 7 (as per Consort Diagram Figure 1). As hypothesised, there was a significant association between percentage of supine sleep overnight and higher pAHI (*r*_s_ = 0.37, *p* = 0.003). However, given there was no difference in percentage of supine sleep during intervention and control nights, there was also no difference in pAHI (intervention = 1.7/h [0.8, 5.4] vs. control = 1.8/h [0.6, 4.4], mean difference = −0.4 [95% CI −1.6, 0.9], *p* = 0.56) or other measures of SDB including pRDI, pODI or snoring (Table 2).

#### 3.3.4 Foetal Heart Rate Analysis

CTG data were available for 47 nights (24 in the control week, 23 in the intervention week, average duration 7.1 ± 2.3 h of successful recording), with paired data across control and intervention nights for 16 women (as per Consort Diagram Figure 1). There were slightly more small decelerations per hour (≥ 10 bpm for ≥ 10 s) on the intervention night (3.0 [1.0]) compared to the control night (2.3 [1.2], mean difference 0.7 [0.1, 1.3], *p* = 0.03; Table 2). This likely represents a chance finding, given that there was no difference in percentage of supine sleep across nights in this sub‐cohort, nor any difference in large decelerations per hour (≥ 20 bpm for ≥ 60 s, likely to be of greater clinical significance) between intervention and control weeks.

From the 47 CTGs available for analysis (independent of intervention or control period), there were 45 one‐hour blocks identified with predominantly supine maternal position (> 50%) versus 281 one‐hour blocks that were predominantly lateral; the average number of foetal heart rate decelerations per hour was not increased during supine sleep compared to lateral sleep (small decels = 1.9 [95% CI 1.4, 2.8] vs. 2.0 [1.6, 2.3], respectively, *p* = 0.92; and large decels = 0.03 [95% CI −09, 0.2] vs. 0.1 [0.06, 0.18], respectively, *p* = 0.14).

#### 3.3.5 Birthweight

To explore associations between objectively confirmed maternal supine sleep and birthweight, the median percentage of supine sleep for every participant (in their control week) was analysed in a cohort manner (as a surrogate for normal sleep behaviour in the third trimester).

The median percentage of supine sleep across the control week was related to lower birthweight and lower birthweight centile (*r*_s_ = −0.45, *p* = 0.007 and *r*_s_ = −0.45, *p* = 0.006, respectively, Figure S2). Gestational age during the control week was not associated with supine sleep (F (1, 33) = 2.28, adj *R* ^2^ = 0.03, *p* = 0.14); however, controlling for gestational age during the control week did attenuate the association between supine sleep and birthweight (F (2, 32) = 3.00, adj *R* ^2^ = 0.11, *p* = 0.069), and birthweight centile (F (2, 32) = 1.79, adj *R* ^2^ = 0.04, *p* = 0.082).

## 4 Discussion

### 4.1 Main Findings

This study has demonstrated that the ‘Back‐Off’ interventional pillow was unable to reliably prevent or reduce the amount of supine sleep overnight during late pregnancy. Women in this study spent approximately 1 h on average per night sleeping on their back in both study arms. As expected, the proportion of supine sleep was significantly associated with SDB. Despite excellent device adherence, the pillow also did not reduce ‘going‐to‐sleep’ in the supine position. Given the intervention pillow did not reduce supine sleep, there was no reduction in SDB severity nor any change in foetal heart rate decelerations overnight. Consistent with our previous study [27], we found an association between the proportion of supine sleep during the control week and infants with a lower birthweight and birthweight centile; however, this relationship was attenuated by gestational age in the control week.

### 4.2 Interpretation

This study commenced after the Safer Baby Bundle [16] national initiative was released in Australia, which includes antenatal education to promote side sleeping after 28 weeks of gestation. We found that 94% of women in this study were aware of safe side‐sleeping practices, with women reporting ‘sleep on left for optimal blood flow to the baby’ and ‘avoid sleeping on my back as it can stress the baby’ and ‘left is best – to sleep on side to encourage best flow of oxygen to baby’. This indicates good uptake of the public health messaging about side sleeping to reduce stillbirth risk. Indeed, recent large surveys have revealed that 90% of pregnant women report receiving advice on the importance of side sleeping, with < 2% reporting they go to sleep on their back [30, 31]. In this study, supine sleep onset was self‐reported on only 0.6% of study nights, despite 8% of nights starting with objectively confirmed supine sleep onset. This was similar to our previous result for supine sleep onset position during uncomplicated pregnancy [10]. We also found that additional pillows at night to support the back were very common in pregnancy, but this did not translate into reduced hours of supine sleep. Despite a concerted effort from women, and those caring for them, to reduce supine sleep, our results suggest that supine sleep onset is underreported, and women spend approximately an hour per night sleeping on their back regardless of pillow support.

The best evidence to date linking going‐to‐sleep position with stillbirth risk has relied on self‐report without validation of maternal position, as methodologically this is a difficult relationship to investigate. These studies may also be prone to recall bias, and this study has shown inconsistencies between self‐reported versus objectively measured sleep onset position. It is uncertain how reflective ‘going‐to‐sleep position’ is of sleep behaviour for the whole night [10], nor how much maternal supine sleep is detrimental to the foetus. Post hoc exploratory analyses suggest a moderately strong association between supine sleep and lower foetal birthweight, highlighting a need to explore this relationship further with larger samples. Two other studies have investigated objectively measured supine sleep during pregnancy and found no association with foetal birthweight— [12, 32] however, both studies relied on a single night of measurement, with one performing the sleep study between 22 and 30 weeks of gestation when the smaller gravid uterus may have fewer effects on uterine blood flow. Alternately, it is possible that supine sleep is avoided in favour of lateral positioning by those with larger foetuses, as they may experience more discomfort lying on the back, or supine hypotension. Given the potential discrepancies between self‐reported and actual sleep behaviour, we suggest that future clinical intervention trials need to objectively record sleep position.

The triple risk model for late pregnancy stillbirth proposes an interplay of maternal and placental/foetal factors along with a stressor [33]. For example, in at‐risk pregnancies, such as those affected by maternal hypertension or obesity, along with poor placental function and abnormal foetal growth, an additional stressor may lead to stillbirth. Supine sleep position and SDB have been proposed as potential acute stressors, given maternal supine position when awake significantly affects blood flow to the uterus and oxygen transfer across the placenta [34], and cerebral redistribution has been associated with reported maternal supine sleep onset position [35]. SDB has been associated with delayed foetal neuromaturation in mothers with obesity [36] and maternal respiratory events have been temporally linked with foetal heart rate decelerations [37]. These stressors may have cumulative effects, as we showed that women who slept more in the supine position on the sleep study night had a higher pAHI, indicating more obstructed breathing.

### 4.3 Strengths and Limitations

Although the sleep position intervention showed no evidence of success, we learned some valuable lessons from this study. Firstly, the huge variability in supine sleep across study nights highlights the importance of obtaining objective measurement from more than one night of sleep, revealing patterns of those consistently side sleeping versus those who should be the focus of interventions. This perhaps explains why some interventions have been successful in single‐night studies but not for longer clinical trials [9, 21, 22]. Secondly, the intervention was well tolerated and did not adversely affect sleep quality. Successful implementation of any device requires comfort and ease of use and this should be considered in the design of future trials.

A limitation of the Night Shift Sleep Positioner was classification of body position into five discrete categories—supine, left, right, prone and upright. One reason we may not have seen an effect of the intervention pillow was that the Night Shift will classify minor abdominal tilt as supine. This is an important distinction to make, as lying fully supine during wake reduces blood pressure, cardiac output and uteroplacental blood flow [38, 39] whereas a lateral tilt of between 15 and 30° may alleviate compression of the inferior vena cava [40], with subsequent elevations in cardiac output [41] and correction of hypotension in some women [42]. Unfortunately, we could not gather information regarding degree of positional rotation from the device, nor was there a commercially available device to measure this at the time of the study. Future studies need to differentiate fully supine from supine with a lateral tilt, to assess the impact of precise sleep positioning on foetal well‐being and intervention effectiveness.

The BMI of participants in this study was largely within the healthy range at the beginning of their pregnancy, which may limit generalisability. A greater impact of supine sleep on SDB and foetal well‐being may be seen in those with a bigger BMI and heavier abdominal wall. We also acknowledge that this cohort was counselled to avoid supine sleep as part of their antenatal education, and therefore, the outcomes could be different in a population without public health policy regarding sleep position in pregnancy.

Lastly, this study was impacted by the COVID‐19 pandemic. The initial study design was to use full polysomnography; however, we needed to change to a device which was self‐applied. Although the WatchPAT uses abbreviated signals, it has been validated in pregnancy with a sensitivity of 0.88 and specificity of 0.87 for detecting mild SDB [26] and was sufficient for a secondary outcome. Several CTG recordings were also impacted, although with instruction several participants were able to successfully apply the foetal heart rate monitor.

## 5 Conclusion

Pregnant women, and those who care for them, are aware of the importance of side sleeping to reduce stillbirth risk. Despite this, supine position at sleep onset is underestimated by self‐report, and we found no evidence to suggest that a device designed to support side sleeping was effective at reducing supine sleep during late pregnancy. This study provides useful insights for the design of future interventional trials, including precise measurement of sleep position, the need for an intervention device that preserves sleep quality and the importance of serial measurements.

## Author Contributions

Danielle L. Wilson involved in conceptualisation, funding acquisition, methodology, project administration, investigation, formal analysis, writing – original draft, writing – review and editing. Carley Whenn contributed to project administration, investigation, formal analysis, writing – review and editing. Maree Barnes contributed to conceptualisation, methodology, writing – review and editing. Susan P. Walker was involved in conceptualisation, funding acquisition, methodology, supervision, writing – review and editing. Mark E. Howard was involved in conceptualisation, funding acquisition, methodology, supervision, writing – review and editing.

## Ethics Statement

The Mercy Hospital for Women Human Research Ethics Committee approved this study on 24 June 2020, project number 2020–015.

## Conflicts of Interest

The authors declare no conflicts of interest.

## Supporting information


Appendix S1.

Figures S1–S2.

## Data Availability

The data that supported the findings of this study are available from the corresponding author upon reasonable request.
