# Supplementary material for: A Position Modification Device for the Prevention of Supine Sleep During Pregnancy: A Randomised Crossover Trial
Source: BJOG. 2024 Sep 16;132(2):145–54. doi: 10.1111/1471-0528.17952 (PMC11625653; doi:10.1111/1471-0528.17952)
Supplement: Supplementary file 1 — Appendix S1. [file BJO-132-145-s002.docx]

Supporting Information – Appendix 1

1. Detailed Method Section

*1.1 Intervention and procedure*

The study design involved two study weeks; 1) the intervention week involved the participant using the “Back-Off” pillow for 7 consecutive nights, and 2) the control week involved the participant using their own pillows for 7 consecutive nights with no additional advice given regarding sleeping position. The participants were block-randomised (in blocks of 4) with half assigned to complete the intervention week first, and the other half assigned to complete the control week first via Study Randomizer.^1^ The first study week was commenced between 30 to 36 weeks of gestation. After a one week ‘wash-out’ period, each participant crossed-over to complete the other arm of the study.

The sleep position modification device was the “Back-Off” pillow (Fig S1), which is a U-shaped pillow designed to prevent supine sleep. One arm of the pillow is soft and designed to support the arms and abdomen, whereas the other arm is firm and rests behind the back, to prevent rolling onto the back. The pillow can be flipped over to use lying on either side. A Velcro strap under the pillow holds the arms together to stop them splaying apart.

On each of the 7 nights of the intervention and control week, the participant’s body position was monitored with the Night Shift Sleep Positioner (Advanced Brain Monitoring, Carlsbad, CA). This match-box sized device is indicated for the treatment of positional obstructive sleep apnoea, and monitors position, movement and sound (for snoring). It is worn at the back of the neck and is held in place with a silicon rubber strap secured by a magnetic clasp. The Night Shift Sleep Positioner is designed to provide vibro-tactile feedback when the supine position is detected; this setting remained off for all participants in this study. A three-axis accelerometer is used to determine neck position and perform an actigraphy-based classification of sleep vs. wake, classified into 30-sec epochs.^2^ Sleep-onset is determined by three consecutive epochs staged as sleep. Neck positions are reported as upright, supine, lateral left, lateral right and prone. Supine is assigned when the neck angle to the left/right is <43 degrees. Lateral left or right is assigned when the neck angle exceeded 47 degrees. The Night Shift Sleep Positioner assumes the participant has remained in the previously assigned position when the neck angle falls between 43 and 47 degrees. Measurement of supine position with the Night Shift Sleep Position is in very close agreement with video inspection.^2,3^

A sleep diary (paper or electronic) was completed each morning, to record perceived sleep duration, sleep quality and sleep position for each night. For the intervention week, questions regarding use and comfort of the Back-Off pillow were included. During the control week questions were asked about use of additional pillows.

On the last night (night 7) of each week, the participants completed an in-home self-applied sleep study with the WatchPAT 300, with concurrent fetal heart rate (cardiotocography; CTG) monitoring using the Monica AN24. The WatchPAT 300 (Itamar Medical, Israel) is a wrist-worn device with a plethysmographic-based finger probe and a sensor adhered to the chest right under the sternal notch, which measures peripheral arterial tone (PAT), oxygen saturation (SpO_2_), heart rate, movement, body position, snoring and chest motion. This device was applied by the participants in their own home, with instructions and a link to a demonstrational video provided. Respiratory events are identified by sympathetic activation with PAT signal amplitude attenuation and heart rate increases, and oxygen desaturations (≥ 3%).^4^ The WatchPat 300 automatic algorithms also use snoring and chest movement to differentiate respiratory event types. PAT Apnoea-Hypopnoea Index (pAHI) was defined as the number of apnoeas and hypopnoeas per hour of sleep. Snoring was evaluated by an acoustic decibel detector in the chest sensor and calculated as the percent of total sleep time with mild snoring >40 dB and moderate snoring >45dB.^5^ Sleep and wake are identified based on patterns of movement from the built-in actigraph. Following the sleep study, recordings were automatically downloaded and analysed in an offline procedure, with respiratory events, sleep stages and snoring level calculated using the proprietary zzzPAT software automated algorithms. The PAT signal is an approved measure for home sleep apnoea testing (HSAT) in the American Academy of Sleep Medicine clinical practice guideline for diagnostic testing for obstructive sleep apnoea.^6^ Among pregnant women, the WatchPat 300 demonstrates excellent sensitivity and specificity for identification of OSA, particularly for pAHI ≥ 5 (sensitivity = 88%, specificity = 87%, AUC = 0.96).^7^

CTG was performed to measure the fetal heart rate using the Monica AN24 (Monica Healthcare Ltd.). The Monica AN24 is a non-invasive portable monitor requiring the placement of 5 adhesive electrodes onto the maternal abdomen to monitor fetal ECG, maternal ECG and uterine EMG to measure uterine contractions and maternal movement. Signal quality is confirmed via Bluetooth signal to the Monica VS software on a laptop. The data are stored in a small unit and were downloaded and scored by the internal algorithm. Each CTG was reviewed by an obstetrician the following day to ensure no clinical action was required. Abnormal CTG warranting notification of the treating physician consist of bradycardia of <100 for >2min, recurrent severe variable decelerations (>1min duration, >2 per 2hr), or repeated late decelerations.^8^

Initially, participants were given the Back-Off pillow, Night Shift Sleep Positioner and WatchPAT300 in person to self-apply at home and were set up with the Monica AN24 by a researcher at the hospital. However, during July to November 2021, equipment was provided to the participants via non-contact drop-offs to their homes due to COVID-19 lockdowns. During this period, researchers were not permitted to meet face-to-face with participants so they were given detailed instructions to attach the Monica AN24 fetal heart rate monitor themselves.

Following completion of both study arms and return of the equipment, participants completed a Sleep Position Questionnaire which included questions on whether they had received any advice regarding sleep position in pregnancy.

Basic demographic and obstetric data were collected at recruitment to the study, including maternal age, parity, gestation and body mass index (BMI) at the first antenatal visit. Birth outcomes included gestation at delivery and birthweight, and birthweight centile was customised for gestational age, maternal height and weight, parity, and fetal sex using the GROW software (Bulk Centile Calculator V8.0.6.2; www.gestation.net).^9^

*1.2 Statistical Analysis*

All statistical analyses were performed with Stata 17.0 (StataCorp LP, College Station, TX). Data are given in means with standard deviation (M (SD)) or median and interquartile range (Mdn [IQR]) for non-normally distributed variables. Mean differences (95% confidence interval) between control and intervention weeks for each of percentage and minutes of supine sleep per night, total sleep time, pAHI and fetal heart rate decelerations were compared to zero (no change) with one-sample t-tests. A two-sided p value of <.05 was considered to indicate statistical significance.

1.2.1 Primary outcome - The primary outcome for this study was the percentage of total sleep time spent in the supine position as measured across each week with the Night Shift Sleep Positioner. Participants who did not complete both study arms were excluded from analysis. The raw data for percentage of TST in the supine position each night across conditions was demonstrated with clustered box plots. To account for the variability across nights within each participant, an intention-to-treat analysis with linear mixed modelling was conducted to investigate nightly percentage of supine sleep in the control and intervention condition across the week. Percentage of supine sleep was log transformed due to positive skewness, then data was back transformed to obtain M ± 95%CI. A mixed model was constructed using control v intervention week and night number (1 to 7) as fixed factors, and a random factor of control v intervention week nested within each participant. Forward stepwise selection was then used to test covariates in the model including BMI, maternal age, parity, gestation, and whether the control or intervention condition was completed first by the participant. The explanatory variable with the smallest p value (of less than 0.20) was added at each step.

1.2.2 Secondary outcomes - Comparison of sleep onset position (supine vs non-supine) was performed with McNemar’s chi square. For comparison of objective and subjective outcome measures of sleep quality across conditions, as measured with the Night Shift Sleep Positioner and Sleep Diary respectively, linear mixed modelling was conducted as described earlier for supine sleep position. Due to skewed data, the following transformations (and subsequent back-transformations) were done – i) sleep efficiency and reported sleep quality was reflected then log transformed, ii) wake after sleep onset (WASO – both objective and subjective), prone %TST and reported sleep latency were log transformed, and iii) reported alertness on waking was reflected then square root transformed.

Comparison of SDB indices on the WatchPAT 300 across control and intervention Night 7 were performed using Wilcoxon signed rank sum tests. To assess the relationship between percentage of supine sleep overnight and SDB, Spearman’s rank order correlation was used due to substantial positive skew in the supine sleep position and pAHI data.

The Monica VS software automatically analyses the fetal heart rate data to produce a parameter table, which includes the percentage of fetal heart rate trace data loss in hourly blocks from the beginning of the recording, along with a tally of the number of fetal heart rate decelerations per hour of trace, classified as small (a decrease in the fetal heart rate from the baseline ≥ 10bpm and lasting for ≥ 10 seconds) and large (a fall in fetal heart rate from the baseline of ≥20 bpm and lasting ≥ 60 seconds). Blocks with >25% fetal heart rate trace data loss were excluded from analysis. Comparison of fetal heart rate decelerations per hour across control and intervention nights were performed using paired-sample t-tests and Wilcoxon signed rank sum tests.

To utilise data from all CTGs, linear mixed model analysis was performed to compare hour blocks of CTG recording containing >50% supine maternal sleep to hour blocks that were predominantly lateral sleep. To do this, maternal sleep position data on the Night Shift Sleep Positioner was synchronized with the CTG recording. The number of small decelerations per hour was log transformed due to positive skew. The mixed model was constructed using supine vs lateral sleep block as a fixed factor, and a random factor of supine vs lateral sleep block nested within each participant.

To investigate the relationship between supine sleep and infant birthweight and birthweight centile, the median percentage of supine sleep for each participant for the control week was analysed in a cohort manner (as a surrogate for normal sleep behaviour in the third trimester). Spearman’s rank order correlation was used due to substantial positive skew in the supine sleep position data. Linear regression was used to adjust for the impact of gestational age on the relationship between supine sleep and birthweight and birthweight centile.

References

1. Study Randomizer [Software Application]. (2017). Available at <http://www.studyrandomizer.com>.

2. Levendowski DJ, Seagraves S, Popovic D, Westbrook PR. Assessment of a neck-based treatment and monitoring device for positional obstructive sleep apnea. J Clin Sleep Med. 2014; 10 (8): 863-871.

3. Levendowski DJ, Veljkovic B, Seagraves S, Westbrook PR. Capability of a neck worn device to measure sleep/wake, airway position, and differentiate benign snoring from obstructive sleep apnea. J Clin Monit Comput. 2015; 29 (1): 53-64.

4. Schwartz A, Schneider H. WatchPAT Scoring Guidelines. Leveraging automated scoring with visual oversight. In: Itamar Medical Ltd. [www.itamar-medical.com](https://uq-my.sharepoint.com/personal/uqdwil27_uq_edu_au/Documents/Documents/D%20Wilson/SDB%20and%20pregnancy%20stuff/Sleep%20Position%20intervention%20manuscript/For%20Submission/BJOG%20revision%20Feb%202024/Reviewed/For%20Submission/www.itamar-medical.com); 2018.

5. Westreich R, Gozlan-Talmor A, Geva-Robinson S, et al. The presence of snoring as well as its intensity is underreported by women. J Clin Sleep Med. 2019; 15 (3): 471-476.

6. Kapur VK, Auckley DH, Chowdhuri S, et al. Clinical Practice Guideline for Diagnostic Testing for Adult Obstructive Sleep Apnea: An American Academy of Sleep Medicine Clinical Practice Guideline. J Clin Sleep Med. 2017; 13 (03): 479-504.

7. O'Brien LM, Bullough AS, Shelgikar AV, Chames MC, Armitage R, Chervin RD. Validation of Watch-PAT-200 against polysomnography during pregnancy. J Clin Sleep Med. 2012; 8 (3): 287-294.

8. Fung AM, Wilson DL, Lappas M, et al. Effects of maternal obstructive sleep apnoea on fetal growth: a prospective cohort study. PLoS ONE. 2013; 8 (7): e68057.

9. Gardosi J, Francis A. Customised Weight Centile Calculator – GROW-Centile v6.6. [www.gestation.net](https://uq-my.sharepoint.com/personal/uqdwil27_uq_edu_au/Documents/Documents/D%20Wilson/SDB%20and%20pregnancy%20stuff/Sleep%20Position%20intervention%20manuscript/For%20Submission/BJOG%20revision%20Feb%202024/Reviewed/For%20Submission/www.gestation.net).
