# Supplementary material for: A Position Modification Device for the Prevention of Supine Sleep During Pregnancy: A Randomised Crossover Trial
Source: BJOG. 2024 Sep 16;132(2):145–54. doi: 10.1111/1471-0528.17952 (PMC11625653; doi:10.1111/1471-0528.17952)
Supplement: Supplementary file 2 — Figures S1–S2. [file BJO-132-145-s001.docx]

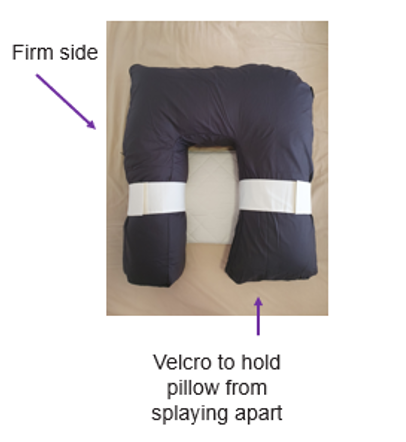


*Figure S1. Back-off Intervention Pillow*


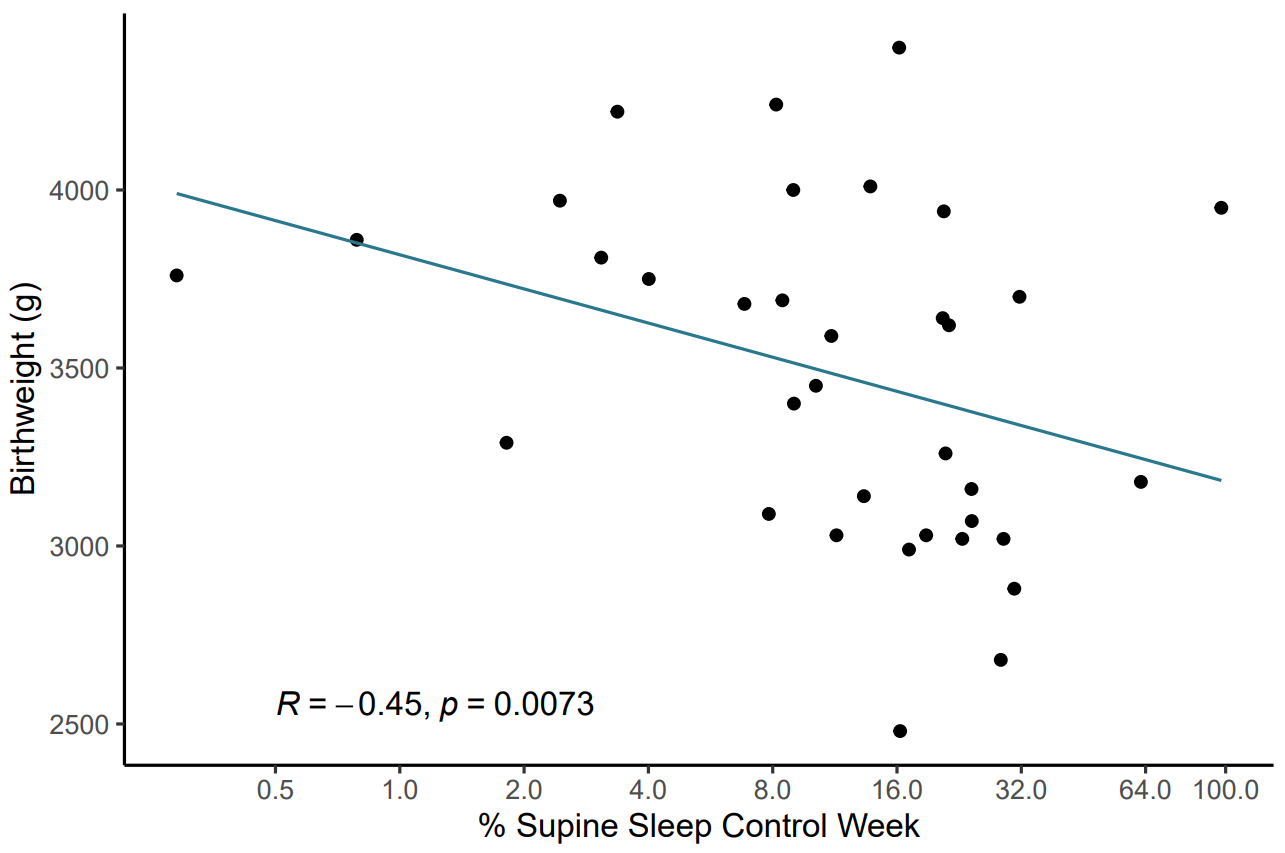


**A**

**B**


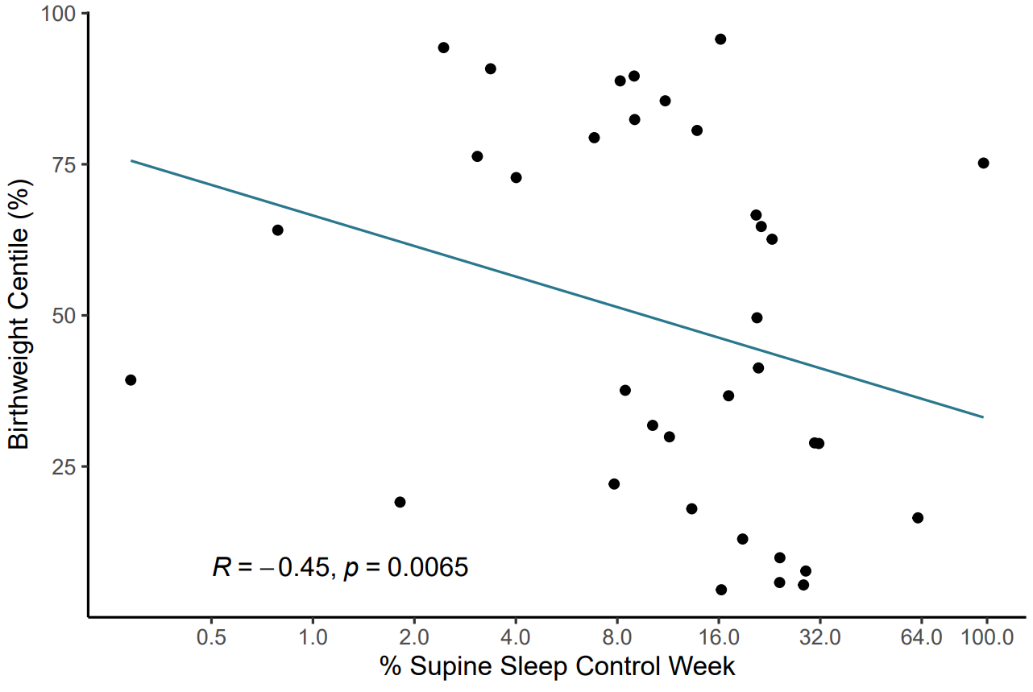


*Figure S2. Scatterplots demonstrating the relationship between the average percentage of supine sleep per night across the control week and A) birthweight and B) birthweight centile. Note: percent of supine sleep is plotted on a log10 scale due to skewness. R = Spearman’s rank order correlation.*
